# Mental Health Status of Healthcare Workers in China for COVID-19 Epidemic

**DOI:** 10.5334/aogh.3005

**Published:** 2020-10-06

**Authors:** Zijun Liu, Jie Wu, Xiuying Shi, Yuhan Ma, Xiao Ma, Zhaowei Teng, Xu You, Yunqiao Zhang, Wenyu Zhang, Ziqiao Feng, Qing Long, Xiaoyuan Ma, Libo Wang, Yong Zeng

**Affiliations:** 1 The Sixth Affiliated Hospital of Kunming Medical University, CN; 2 Yunnan Mental Health Center, CN; 3 Yuxi People’s Hospital, CN; 4 Yuxi Second People’s Hospital, CN

## Abstract

**Background:**

COVID-19 first appeared in China in December 2019, with a high rate of infectivity and morbidity, which brought tremendous psychological pressure to healthcare workers.

**Purpose:**

To understand the psychological health status of healthcare workers during the COVID-19 outbreak and decline, and to provide a theoretical reference for the future establishment of a psychological crisis intervention system.

**Methods:**

Healthcare workers were recruited using convenience sampling and snowball sampling methods, and the electronic version of the SCL-90 scale and a sociodemographic questionnaire were administered. In the pretest, a total of 5018 responses were collected; after six weeks, random sampling was performed. The SCL-90 and measures of other epidemic-related problems were administered, with 1570 responses received; then, the final data analysis was performed.

**Results:**

After six weeks, the post-test GSI score; SCL-90 total score; and PST, PSDI, O-C, I-S, DEP, ANX, PHOB, PAR, PSY, and HOS scores were significantly lower than the corresponding pretest scores (p < 0.05). The results by occupational category showed that the scores of nursing staff decreased significantly for 12 indexes and that the scores of the doctors and other hospital staff also significantly decreased. There was a significant difference between the pretest (50.78 ± 28.18) and post-test (45.00 ± 28.49) scores for the degree of worry about the epidemic. Healthcare workers believed that the top three aspects of life affected by the epidemic were economic problems (816 people), interpersonal communication problems (731 people), and mental health (728 people).

**Conclusion:**

Over the course of the epidemic, the item scores generally declined significantly. Therefore, during an outbreak period, attention should be paid to psychological crisis interventions for healthcare workers; problems caused by psychological pressure, and even other psychological conditions, can be significantly alleviated to reduce the probability of subsequent health problems.

## 1. Background

Coronavirus disease 2019 (COVID-19) is a new infectious disease caused by SARS-CoV-2, with epidemiological characteristics such as strong infectivity, high morbidity, multiple infection routes, and widespread infection [1,2]. Since the epidemic began in Wuhan, China, in December 2019, from January 2020 to April 2020, COVID-19 has spread explosively in China. As of May 24, a total of 5,290,506 cases had been diagnosed worldwide, with a total of 342,448 deaths. A total of 84,525 cases had been diagnosed in China, with 4,645 deaths and 79,749 cured [3]. The spread of the COVID-19 epidemic occurred exceptionally quickly, and its range is extensive, covering almost all countries in the world. Since the outbreak in China in December, the outbreak has been effectively controlled through the development of the Chinese government’s prevention and control measures. On February 25, 2020, there were no new cases in 26 provinces across the country. The Chinese government began to gradually ease the controls of the epidemic situation and initiate the national resumption of production and orderly recovery, and China’s anti-epidemic efforts have achieved effective results. Health workers are among the most important people in every major public health challenge, as frontline anti-epidemic workers, have made great contributions to anti-epidemic work and have experienced great psychological pressure, which may increase the current baseline level of psychopathology [4], it can even lead to related disorders, stress, anger, and mood dysregulation [5], can not continue to put into work. Therefore, the mental health issues of healthcare workers cannot be ignored. On January 26, 2020, the National Health Commission of China issued the notification of principles for emergency psychological crisis intervention for the COVID-19 epidemic. Local governments responded actively, establishing a series of psychological crisis intervention systems based on internet intervention. At present, there is still a lack of investigation and comparative research on healthcare workers’ mental health statuses before and after the outbreak. We followed up healthcare workers six weeks and compared their mental health status before, and after the outbreak; therefore, this article aims to play an auxiliary role in adjusting the psychological intervention strategy in the face of major public emergencies in the future.

## 2. Methods and Subjects

### 2.1 Sampling methods

Convenience sampling, snowball sampling, random sampling. Because the SARS-CoV-2 virus is extremely contagious, so use the Internet to collect questionnaires. For the pretest, media promotion and in-hospital promotion methods were used to randomly select healthcare workers from different hospitals across the country; then, the study was further promoted and publicized via each hospital’s healthcare workers, thereby expanding the number and scope of subjects. For the post-test, people who had completed the pretest questionnaires were randomly selected to complete the online questionnaire and post the link to the questionnaire publicly to recruit additional participants. The first test was implemented from January 29 to February 3, 2020, which was during the epidemic outbreak period in China; the second test was implemented from March 13 to March 18, 2020, during which time the epidemic was declining [6], and the interval between two tests were approximately six weeks.

### 2.2 Subjects

This study included healthcare workers (including doctors, nursing staff, and other hospital staff) in mainland China who were working in medical positions from January 2020 to April 2020 and were most likely to be exposed to suspected or confirmed cases of COVID-19 during the fight against the epidemic. The hospitals where these participants worked were designated COVID-19 admission hospitals, and the departments where they worked were usually hot and treated many emergencies, respiratory, and severe ICU patients.

The research subjects included Chinese citizens over 16 years old. A total of 5258 questionnaires were distributed in the pretest, and a total of 5018 questionnaires were returned, for an effective rate of 95.43%. A total of 1722 questionnaires were distributed in the post-test, and a total of 1570 valid questionnaires were returned, for an effective rate of 91.17%. A total of 1340 participants in the pretest also returned effective post-test questionnaire responses, accounting for 85.35% of the post-test participants; the number of new participants in the post-test was 230, accounting for 14.65% of the post-test participants.

### 2.3 Investigation method: Self-report sociodemographic questionnaire and the Symptom Check List-90

An online questionnaire survey using the world-recognized Symptom Check List-90 (SCL-90) [7,8,9,10], and sociodemographic items related to the epidemic situation was adopted. The SCL-90 includes nine factors that reflect various psychological symptoms of the individual: Somatization (SOM), Obsessive-Compulsive disorder (O-C), Interpersonal Sensitivity (I-S), Depression (DEP), Anxiety (ANX), Anger-Hostility (HOS), Phobic Anxiety (PHOB), Paranoid Ideation (PAR), and Psychoticism (PSY). In addition, a Global Severity Index (GSI), Total Score (T-S), Positive Symptom Total (PST) and Positive Symptom Distress Index (PSDI) can be calculated, for a total of 13 comprehensive assessments of mental health levels. The scale consists of a total of 90 assessment items; respondents indicate their responses to each item on a five-point scale from 0–4, corresponding to “not at all”, “a little bit”, “moderately”, “quite a bit” and “extremely”. The higher the score of the surveyed individual, the greater his or her mental health conforms to the description of the symptom, which indicates a worse mental health level. The instructions presented before the questionnaire and the instructions for informed consent were both provided electronically and were collected and screened after the questionnaire evaluation. The answers to the pretest and post-test questionnaires contained approximately 2000 words; based on this level of difficulty of the text, the estimated reading speed for the text was 10–50 words per second. Based on previous research, we expected that a completion speed greater than or equal to 150 seconds would be acceptable for the questionnaires. Therefore, questionnaires completed in less than 150 seconds or contained inconsistent answers were not included in the statistical analysis.

### 2.4 Statistical methods and indicators

The SPSS 22.0 statistical software package was used for data analysis. For the SCL-90 scale; the PST was calculated as the total number of items with a score greater than 0; the PSDI was calculated as the total questionnaire score divided by the PST; the GSI, which is a very effective score to reflect the results [11], was calculated as the total score of the questionnaire divided by 90; and the T-S was calculated as the sum of the scores of each item. The scores for the remaining nine factors were obtained by dividing the total score of the items for each factor by the number of items for each factor. In the sociodemographic questionnaire, the scores for the degree of worry about the epidemic, the evaluation of the epidemic psychological crisis intervention system, and satisfaction with the government’s response measures ranged from 0–100 points.

## 3. Results

### 3.1 General sociodemographic information

A total of 5018 valid questionnaires were collected in the pretest. The age range of the participants was 16–64 years old; participants born in 1980–1989 constituted the largest age group, accounting for 36.6% of the sample. The total gender composition was mainly female, with females accounting for 80.8% of the sample. The participants mainly had college or undergraduate education levels, with these participants accounting for 88.5% of the sample. Regarding marital status, most of the participants were married, accounting for 73.6% of the sample. The pretest included participants from Wuhan and other cities, and frontline anti-epidemic personnel accounted for 49.3% of the sample. Doctors, nursing staff, and other staff of the hospital accounted for 32.7%, 53.0%, and 14.3% of the sample, respectively. A total of 1570 valid questionnaires were collected in the post-test. The age range was between 18–64 years old. Participants born after the 1980s were again dominant, accounting for 46.1% of the sample. Women accounted for 80.3% of the sample. First-line anti-epidemic healthcare workers accounted for 37.3% of the sample, while doctors, nursing staff, and other staff in the hospital accounted for 27.8%, 51.0%, and 21.1%, respectively; see Table 1 for details.

**Table 1:** Comparison of participants’ general sociodemographic characteristics.

	Demographics	Pretest	Posttest	Total

Gender	Male	964	310	1274
	Female	4054	1260	5314
Education level	Primary school	2	2	4
	Junior high school	36	7	43
	High school	212	43	255
	Undergraduate	4439	1400	5839
	Master’s degree	286	117	403
	Doctor of Philosophy	43	1	44
Marital status	Unmarried	1197	282	1479
	Married	3690	1236	4926
	Divorced	114	44	158
	Widowed	17	8	25
Occupation	Doctor	1642	437	2079
	Nursing staff	2658	801	3459
	Other hospital staff	718	332	1050
Work position	Frontline	2474	585	3059
	Non-frontline	2544	985	3529
Birth year	After 1950	12	3	15
	After 1960	441	131	572
	After 1970	934	336	1270
	After 1980	1837	724	2561
	After 1990	1777	375	2152
	After 2000	17	1	18
	Mean ± standard deviation	34.46 ± 9.27	35.68 ± 8.54	34.74 ± 9.07

### 3.2 Comparison of pre- and post-test SCL-90 scores

From the pretest to the posttest, all healthcare workers the GSI, PST, PSDI, and T-S all decreased significantly. The SOM score showed a little increase from the pretest, while the O-C, I-S, DEP, ANX, HOS, PHOB, PAR, and PSY scores all presented significant decreases. Among the scores, the O-C pretest score of 0.45 ± 0.54 and the posttest score of 0.40 ± 0.53 are the highest scores among the factors. The ANX score showed the largest difference between the pre- and posttest. The healthcare workers were divided into groups by occupation, and the doctor group showed significant decreases between the pre- and posttest in both their ANX (0.26, 0.20, p < 0.01) and PSDI (1.19, 1.07, p < 0.01) scores. Nursing staff showed significantly decreased scores on the following 12 indexes: OC (0.53, 0.44, p < 0.01), IS (0.37, 0.28, p < 0.01), DEP (0.33, 0.27, p < 0.01), ANX (0.36, 0.24, p < 0.01), HOS (0.32, 0.27, p < 0.01), PHOB (0.35, 0.26, p < 0.01), PAR (0.25, 0.19, p < 0.01), PSY (0.22, 0.17, p < 0.01), TS (30.33, 24.93, p < 0.01), PST (19.93, 17.60, p < 0.01), GSI (0.0.34, 0.28, p < 0.01), PSDI (1.26, 1.11, p < 0.01). Other hospital staff had significantly decreased scores for ANX (0.26, 0.21, p < 0.05) and PSDI (1.19, 1.09, p < 0.01). ANX scores decreased significantly among all three groups of personnel. See Table 2 and Figure A for details.

**Table 2:** Comparison of the pre- and posttest SCL-90 scores of healthcare workers (x̅ ± s).

Factor	All healthcare workers			Doctors			Nursing staff			Other hospital staff		

	Pre	Post	p	Pre	Post	p	Pre	Post	p	Pre	Post	p

GSI	0.29 ± 0.40	0.26 ± 0.39	0.001	0.24 ± 0.36	0.23 ± 0.40	0.479	0.34 ± 0.43	0.28 ± 0.20	0.000	0.25 ± 0.36	0.24 ± 0.33	0.767
PST	17.70 ± 19.39	16.33 ± 20.75	0.016	14.96 ± 18.12	14.21 ± 19.50	0.454	19.93 ± 20.30	17.60 ± 22.06	0.005	15.71 ± 17.46	16.03 ± 18.79	0.785
PSDI	1.36 ± 0.42	1.10 ± 0.60	0.000	1.19 ± 0.57	1.07 ± 0.61	0.000	1.26 ± 0.56	1.11 ± 0.60	0.000	1.19 ± 0.55	1.09 ± 0.57	0.004
T-S	26.39 ± 35.60	23.03 ± 34.82	0.001	21.82 ± 32.37	20.55 ± 35.75	0.479	30.33 ± 38.29	24.93 ± 36.28	0.000	22.30 ± 30.24	21.71 ± 29.40	0.767
SOM	0.22 ± 0.37	0.23 ± 0.40	0.427	0.18 ± 0.33	0.19 ± 0.38	0.599	0.26 ± 0.40	0.27 ± 0.44	0.606	0.18 ± 0.31	0.20 ± 0.34	0.519
O-C	0.45± 0.54	0.40 ± 0.53	0.001	0.36 ± 0.48	0.34 ± 0.53	0.459	0.53 ± 0.57	0.44 ± 0.55	0.000	0.39 ± 0.47	0.39 ± 0.48	0.825
I-S	0.32 ± 0.48	0.27 ± 0.43	0.000	0.27 ± 0.44	0.25 ± 0.44	0.528	0.37 ± 0.52	0.28 ± 0.45	0.000	0.27 ± 0.41	0.26 ± 0.37	0.607
DEP	0.30 ± 0.47	0.26 ± 0.44	0.002	0.26 ± 0.44	0.24 ± 0.47	0.547	0.33 ± 0.49	0.27 ± 0.45	0.001	0.25 ± 0.41	0.24 ± 0.40	0.648
ANX	0.31 ± 0.46	0.22 ± 0.41	0.000	0.26 ± 0.41	0.20 ± 0.42	0.005	0.36 ± 0.50	0.24 ± 0.43	0.000	0.26 ± 0.40	0.21 ± 0.34	0.035
HOS	0.27 ± 0.45	0.24 ± 0.43	0.008	0.22 ± 0.41	0.20 ± 0.44	0.369	0.32 ± 0.49	0.27 ± 0.46	0.010	0.22 ± 0.37	0.21 ± 0.34	0.873
PHOB	0.31 ± 0.47	0.25 ± 0.42	0.000	0.26 ± 0.43	0.26 ± 0.43	0.289	0.35 ± 0.50	0.26 ± 0.44	0.000	0.27 ± 0.42	0.25 ± 0.37	0.503
PAR	0.22 ± 0.39	0.19 ± 0.36	0.006	0.18 ± 0.36	0.18 ± 0.39	0.870	0.25 ± 0.43	0.19 ± 0.37	0.001	0.18 ± 0.33	0.18 ± 0.32	0.782
PSY	0.19 ± 0.37	0.16 ± 0.35	0.001	0.15 ± 0.34	0.15 ± 0.38	0.684	0.22 ± 0.40	0.17 ± 0.35	0.000	0.17 ± 0.32	0.15 ± 0.30	0.587

**Figure:**
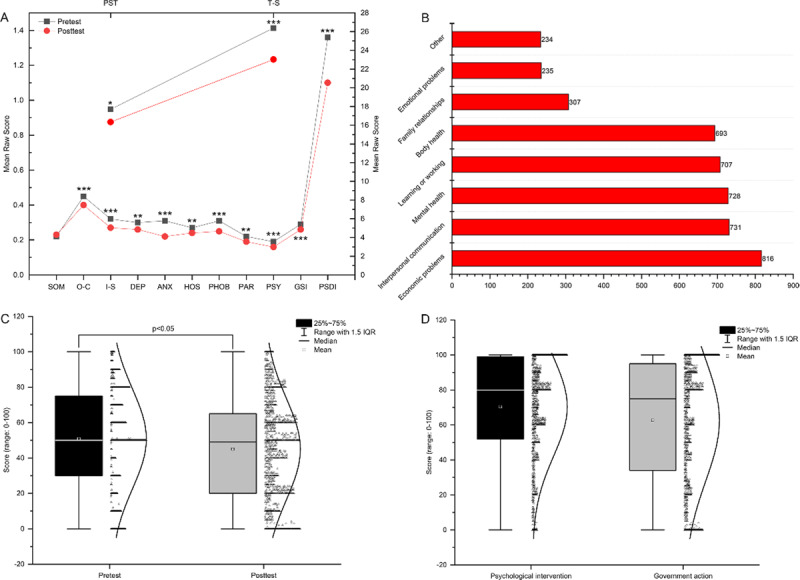
**(A)** Comparison of all healthcare workers pretest and post-test SCL-90 scores. **(B)** Impact of the epidemic on life. **(C)** Pretest and post-test scores for the degree of worry about the epidemic. **(D)** Scores for the evaluation of the psychological intervention system and the degree of satisfaction with government.

### 3.3 Multiple logistic regression analysis of the influence of different factors on the total mental health score

Gender, age, marital status, education level, occupation, frontline healthcare worker status and SCL-90 total score were analyzed in the multiple logistic regression analysis; gender (OR = 0.80, p < 0.05), age (OR = 0.99, p < 0.01), whether frontline healthcare (OR = 0.59, p < 0.01) were all independent protective factors as indicated by the omnibus test of the regression model (p < 0.01). The observed Hosmer-Lemeshow goodness of fit value was 4.49 (p = 0.811), and the degree of fit was high. See Table 3 for details.

**Table 3:** Multivariate logistic regression analysis of different factors on the total score.

Factor	B	SE	Wald X^2^	p	OR	95% CI

Gender	–0.23	0.11	4.12	0.04	0.80	0.637–0.992
Age	–0.01	0.01	6.01	0.01	0.99	0.980–0.998
Marital status	0.09	0.09	1.08	0.30	1.10	0.922–1.302
Education level	0.03	0.11	0.07	0.79	1.03	0.831–1.274
Occupation	–0.12	0.07	3.13	0.08	0.89	0.780–1.013
Work position	–0.53	0.08	39.14	0.00	0.59	0.502–0.697
Constant	–1.04	0.55	3.64	0.06	0.35	

### 3.4 Impact of the epidemic on life

The aspect of life most strongly affected during the epidemic was economic problems; 52% of the total sample selected this option. The next most commonly selected option was interpersonal communication problems, at 46.6%, followed by mental health issues, at 46.4%; learn or work, at 45.0%; body health issues, at 44.1%; family relationship issues, at 19.6%; emotional issues, at 15.0%; and other issues, at 15.0%. See Figure B for details.

### 3.5 Comparison of the degree of worry before and after the epidemic

Both the pretest and post-test questionnaires asked, “How worried are you about this epidemic?” A 0–100 rating scale is used, where 0 points indicated not worried at all, and 100 points indicated the participant was worried about panic. In the pretest, most of the scores were concentrated at the high end of the scale, with a median score of 50 and an average score of 50.78 ± 28.18. In the post-test, the scores were more uniform; the number of scores at the low end of the scale increased, with a median score of 50 and an average score of 45.00 ± 28.49. The difference between the pretest and post-test worry scores was significant (p < 0.01). There was also a strong correlation between the degree of anxiety about the epidemic and the SCL-90 total score (r = 0.305); see Table 4 and Figure C for details.

**Table 4:** Posttest outbreak worry score.

Factor	Pretest	Posttest	p	95% CI (lower-higher)	r		

Mean	50.78 ± 28.18	45.00 ± 28.49	0.000	4.181–7.384	0.305**

### 3.6 COVID-19 epidemic healthcare workers’ subjective evaluations of the psychological intervention system during the epidemic period

During the epidemic, 81.5% of people reported an excellent evaluation of the psychological intervention, with a median score of 80 and an average score of 70.37 ± 28.31; the median score for overall satisfaction with the measures taken by the government during the epidemic was 78, and the average value was 62.65 ± 34.77, see the Figure D for details.

## 4. Discussion

### 4.1 Post-test were significantly lower than the pre-test results

The two assessments carried out during the epidemic were conducted during the period when the epidemic was typical. The pretest was conducted at the end of January when the outbreak was severe. The post-test was conducted in the middle of March, six weeks later. Some studies have shown that after a crisis, psychological crisis intervention should be carried out as soon as possible and that crisis intervention carried out after six weeks will have little effect [12]. Only 30% of patients will recover on their own without intervention, 40% of patients will have mild symptoms, and 10% of patients will even worsen over time [13]. This study post-test scores decreased significantly, we believe that these two evaluations can evaluate the effectiveness of the psychological crisis intervention system during the epidemic. Data from frontline healthcare workers in the early HIV epidemic, SARS, Ebola, and other epidemics demonstrates that working at the frontline of a epidemic has significant psychiatric repercussions including burnout, anxiety and posttraumatic stress disorder [11,12,13,14,15]. The most representative score, i.e., the GSI, significantly decreased between the pretest and post-test. Except for the SOM score, all SCL-90 scores were significantly reduced, indicating that the psychological crisis intervention system during the epidemic had a positive psychological effect on healthcare workers. The logistic regression analysis showed that men and younger, non-frontline workers had higher mental health levels than the other participants, indicating that the pressure of frontline anti-epidemic work is very high and is more likely to affect the total mental health score. During the epidemic, the PHOB score is very high in the pretest and drops significantly in decline; the O-C factor had the item with the highest score because COVID-19 is extremely contagious, practices such as repeated hand washing and work inspections have become protective factors, reducing the chance of infection of healthcare workers; SOM was the only factor that had a higher post-test score than pretest score. It is speculated that healthcare workers are highly stressful and that work pressure during an outbreak will contribute to changes in the immune system, endocrine system, [12], and maybe cause severe stress disorder [16,17]. Posttraumatic stress disorder (PTSD) symptoms include severe ANX symptoms [18]. The post-test scores showed a significant decrease in ANX scores, which may indicate an improvement in stress conditions because the mental state is relaxed during the decline epidemic. Hence, SOM symptoms such as headaches, chest tightness, and muscle aches begin to appear. To identify which participants’ scores decreased more significantly, the healthcare workers were divided into three groups, i.e., doctors, nursing staff, and other hospital staff, and the nursing staff had the highest pretest score overall, followed by other hospital staff, and finally doctors. During the anti-epidemic process, compared with the other two groups, the nursing staff had the closest contact with the patient, and their work was more burdensome than that of the other groups. Therefore, nurses’ pretest scores for various symptoms were higher. Epidemics may cause collective fear and impose lifestyle changes for all peoples, not only those directly impacted by infection [19], and the lifestyle of healthcare workers is also affected by many aspects.

### 4.2 Psychological intervention measures for healthcare workers during the epidemic

During the epidemic, China adopted a series of psychological intervention measures for healthcare workers, which possibly contributed to the significant reductions in the scores of healthcare workers from the pretest to post-test. Data from the H1N1 influenza epidemic in Japan showed that institutional trust was correlated with motivation to work at the frontline, we can engender feelings of institutional trust by demonstrations of genuine support for our colleagues wellbeing and mental health [20]. So during the epidemic, various provinces and cities established 24-hour psychological assistance hotlines in response to the crisis intervention notice [21], Each hospital had at least one psychological crisis intervention team to support the intervention. In addition, online group intervention and screening for serious psychological problems were provided. Regarding psychological intervention, healthcare workers could also make psychological crisis intervention calls anonymously to alleviate psychological problems. For healthcare workers participating in the frontline anti-epidemic efforts, financial subsidies and special family care were provided to alleviate worries. In addition, a centralized occupation change system was implemented, after multiple days of concentrated work, an isolation period of 14 days was established. During this period, psychological screening and intervention were conducted to ensure that the work pressure of healthcare workers was reduced as much as possible. Therefore, with the help of psychological intervention, proper recognition, and adaptation to the work rhythm, the level of mental health also increased significantly.

### 4.3 Effective measures taken by China during the epidemic

During the epidemic, a series of measures to protect national security was proposed. First, closures were implemented at the community for at least two weeks throughout the country, and registration was required for entry and exit, which reduced the flow of people and made the destination of travel clear. Second, media propaganda reduced panic and normalized people’s emotional cognition. The popularization of digital technology and smart devices provided great help for medical information propaganda [22]. Studies have shown that the satisfaction and treatment effect generated by telemedicine services is very high [23,24]. Therefore, the use of network psychological intervention is effective and safe. In addition, free medical treatment for diagnosed patients has allowed an increasing number of people to attempt to treat the disease and gain the courage to conduct testing, reducing the potential risk of infection due to refusing to see a doctor for economic reasons; ultimately, the suspected individual isolation at home further reduced the risk of cross-infection. These measures give the public a sense of security, which is the cornerstone of maintaining national stability. Hospitals adopted special fever treatment channels, active donations of materials across society were organized, and the supply of materials to frontline healthcare workers was prioritized, which all reduced the risk of cross-infection and ensured the personal safety of healthcare workers. Healthcare workers have strong support, and the safety of the public is supported by healthcare teams. Therefore, all participants’ satisfaction with the government’s measures reached 62.65 points, and the average satisfaction with the psychological intervention system was as high as 70.37 points. Since the public has been highly cooperative, China is controlling the epidemic very quickly.

## 5. Conclusion

This study shows that the level of worry about the epidemic is significantly related to the level of mental health, so the measures taken by the government are very important. When the public feels more secure, they will not experience feelings of uncertainty about the epidemic, which can lead to a decline in anxiety and improve mental health. There was a large difference between the mental health level of healthcare workers during the outbreak and during the decline of the epidemic. During outbreaks, more attention should be paid to the mental health status of healthcare workers, and nursing staff should be given more attention than workers with other occupations. After a psychological crisis intervention during an outbreak, most healthcare workers’ mental health levels will quickly return to normal levels, and some prework in the early stage is effective. In the later stage, psychological intervention is needed only for individuals who do not experience relief in work pressure, which is helpful to alleviate long-term distress after the epidemic, prevent chronic PTSD and physical disease.
